# Clinical predictors of left ventricular thrombus after myocardial infarction as detected by magnetic resonance imaging

**DOI:** 10.3389/fcvm.2023.1275390

**Published:** 2024-01-16

**Authors:** Alexander Fardman, Eias Massalha, Sharon Shalom Natanzon, Yafim Brodov, Orly Goitein, Fernando Chernomordik, Romana Herscovici, Avishay Grupper, Rafael Kuperstein, Israel Mazin, Shlomi Matetzky, Roy Beigel

**Affiliations:** ^1^ The Cardiovascular Division, Sheba Medical Center, Tel-Hashomer, Affiliated to The Sackler Faculty of Medicine, Tel-Aviv University, Tel Aviv, Israel; ^2^ Department of Cardiology, Cedars Sinai Heart Institute, Los Angeles, CA, United States

**Keywords:** STEMI, left ventricular thrombus, ThrombScore, prediction, CMRI

## Abstract

**Background:**

The diagnosis of a left ventricular (LV) thrombus in patients with ST-segment elevation myocardial infarction (STEMI) remains challenging. The aim of the current study is to characterize clinical predictors for LV thrombus formation, as detected by cardiac magnetic resonance imaging (CMRI).

**Methods:**

We retrospectively evaluated 337 consecutive STEMI patients. All patients underwent transthoracic echocardiography (TTE) and CMRI during their index hospitalization. We developed a novel risk stratification model (ThrombScore) to identify patients at risk of developing an LV thrombus.

**Results:**

CMRI revealed the presence of LV thrombus in 34 patients (10%), of whom 33 (97%) had experienced an anterior wall myocardial infarction (MI), and the majority (77%) had at least mildly reduced left ventricular ejection fraction (LVEF < 45%). The sensitivity for thrombus formation of the first and second TTE was 5.9% and 59%, respectively. Multivariate logistic regression model revealed that elevated C-reactive protein levels, lack of ST-segment elevation (STe) resolution, elevated creatine phosphokinase levels, and STe in anterior ECG leads are robust independent predictors for developing an LV thrombus. These variables were incorporated to construct the ThrombScore: a simple six-point risk model. The odds ratio for developing thrombus per one-point increase in the score was 3.2 (95% CI 2.1–5.01; *p* < 0.001). The discrimination analysis of the model revealed a c-statistic of 0.86 for thrombus development. The model identified three distinct categories (I, II, and III) with corresponding thrombus incidences of 0%, 1.6%, and 27.6%, respectively.

**Conclusion:**

ThrombScore is a simple and practical clinical model for risk stratification of thrombus formation in patients with STEMI.

## Graphical Abstract

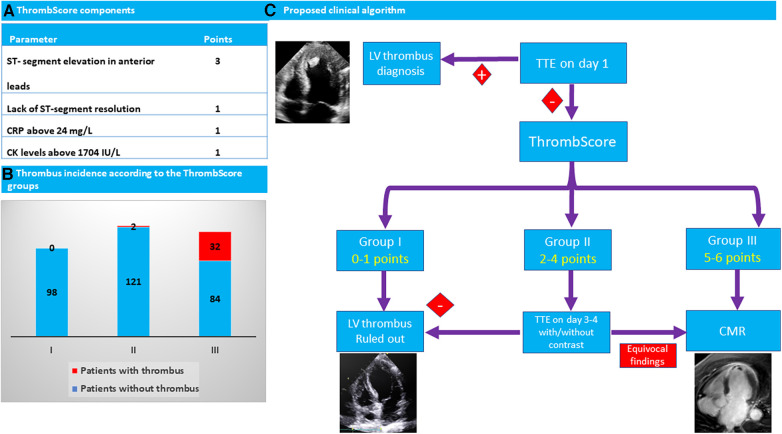

(**A**) Four parameters were found to be independently associated with left ventricular (LV) thrombus development and were incorporated to construct the ThrombScore: a simple six-point risk model. (**B**) ThrombScore identified three distinct risk categories (I, II, and III) with corresponding thrombus incidences of 0%, 1.6%, and 27.6%, respectively. (**C**) Clinical algorithm is proposed based on imaging and ThrombScore. In each STEMI patient hospitalized, TTE is performed on the first hospitalization day. In patients with a negative TTE, the ThrombScore should be assessed. In patients within the lowest (I) category (0–1 points), an LV thrombus can be safely ruled out. In patients within the highest (III) category (5–6 points), CMRI is recommended to rule out an LV thrombus. Patients within the intermediate (II) category (2–4 points) should probably undergo re-evaluation with TTE with or without contrast and be considered for imaging by CMRI on an individual basis. CMRI, cardiac magnetic resonance imaging; CK, creatine phosphokinase; CRP, C-reactive protein; LV, left ventricular; TTE, transthoracic echocardiography; STe, ST-segment elevation; STEMI, ST-segment elevation myocardial infarction.

## Introduction

Left ventricular (LV) thrombus formation is a concerning complication of ST-segment elevation myocardial infarction (STEMI) due to the risk of devastating thromboembolic events. The incidence of LV thrombus has declined significantly since the introduction of primary percutaneous coronary intervention (PPCI) and advancements in peri-procedural care for STEMI patients; however, the reported rates of LV thrombus vary (1). Several studies relying on transthoracic echocardiographic (TTE) evaluation have found rates of 0.4%–2.7% in STEMI patients in general, and up to 9% in those with anterior STEMI. However, a contemporary meta-analysis of studies utilizing cardiac magnetic resonance imaging (CMRI) imaging has reported an incidence rate of up to 6.3% in STEMI patients, which increases to 19.2% when specifically evaluating those with anterior STEMI and reduced LV systolic function [left ventricular ejection fraction (LVEF) < 50%] (2–4). This discrepancy in the incidence of an LV thrombus could be explained by the different imaging modalities used in each study, as previous studies have described significantly lower sensitivity and negative predictive value of echocardiography, as compared with CMR imaging, for the detection of an LV thrombus, thereby suggesting that the true incidence may be underestimated when relying solely on TTE (4–7). As the routine use of CMRI for every STEMI patient is not feasible, additional predictors are needed to better stratify which STEMI patients are prone to develop an LV thrombus and are in need of a more in-depth evaluation.

The aim of the current study is to define predictors for the development of an LV thrombus as diagnosed by CMRI in a contemporary cohort of patients without a prior history of coronary artery disease (CAD) presenting with a STEMI and undergoing primary percutaneous intervention.

## Methods

### Patient characteristics

We conducted a retrospective analysis of the medical records of 337 consecutive patients who presented with their first STEMI, had no previous diagnosis of coronary artery disease, and were referred to CMRI during their hospitalization stay. STEMI was diagnosed in compliance with the contemporary guidelines for STEMI management set by the European Society of Cardiology (ESC) and American College of Cardiology (ACC) (8, 9). All study participants underwent PPCI and were treated with dual antiplatelet therapy (DAPT) based on the physician's discretion and in compliance with current guidelines (8, 9).

Demographic characteristics, comorbidities, pain to balloon time, electrocardiographic (ECG) findings including myocardial infarction (MI) location, and $\Sigma$ST-segment elevation (STe) at presentation and upon the first ECG post-primary PCI were prospectively documented. STe resolution was defined as at least 70% reduction in the STe at ECG 1 h post-PCI (9). All ECGs were reviewed by two cardiologists (AF and EM).

All patients underwent at least one TTE during hospitalization. TTE was performed by an experienced sonographer using commercial equipment. Images were acquired in standard orientations according to recommendations from the American Society of Echocardiography (10). During the study period, the Troponin-I assay changed from Access AccuTnI (Beckman Coulter, Inc.; positive TnI was defined as greater or equal than 0.07 μg/L) to highly sensitive Troponin-I ACCESS hsTnI (Beckman Coulter, Inc.; positive TnI was defined as greater or equal to 12 ng/L in women and 20 ng/L in men). Thus, we divided troponin levels to tertiles according to the assay used. The study was approved by the Institutional Review Board of the Sheba Medical Center (SMC-8939-21).

### CMRI analysis

All patients underwent CMRI on day 5 ± 2 following admission. All scans were performed using either a 1.5-Tesla scanner (General Electric, Optima mr450w GEM versionDV26) or a 3-Tesla scanner (Philips Ingenia 3T version 5.4.1.2), according to scanner availability. The scans were performed using the following sequences: steady-state free precession (SSFP) (short axis, four-chamber, two-chamber, and three-chamber planes) and late gadolinium enhancement (LGE) (short axis, four-chamber, two-chamber, and three-chamber planes). Typical SSFP acquisition parameters were as follows: TR/TE = 3/1, flip angle = 45°, in-plane resolution: 1.7*1.7, slice thickness = 8 mm for 1.5T scanner; TR/TE = 4.3/2, flip angle = 25°, slice thickness = 8 mm for 3T scanner. LGE was collected 10–15 min after the administration of 0.1 mmol/kg contrast agent (Gadoterate meglumine, Guerbet S.A., France). The inversion time was adjusted for optimal nulling of remote normal myocardium.

### Thrombus identification

The LV thrombus was identified upon TTE using standard anatomic criteria: an echodense mass within the LV cavity adjacent to a hypokinetic or akinetic myocardium with margins distinct from the endocardium and distinguishable from other intracavitary objects such as papillary muscles, chordae, trabeculations, or artifacts. Upon CMRI, the thrombus was detected by LGE using a post-contrast segmented inversion-recovery sequence. The LV thrombus was characterized as a low-signal intensity mass surrounded by high-signal intensity structures such as intracavitary blood and/or hyper-enhanced myocardial scar (11, 12).

### Statistical analysis

Categorical variables are reported in frequencies and percentages and compared using the chi-square test or Fisher's exact test. Normally distributed continuous variables were reported as mean and standard deviation values, and differences between groups were assessed using the Student's *t*-test. Continuous variables not normally distributed were reported as median and interquartile range (IQR, 25th–75th percentiles) values, and significance was assessed using the Mann–Whitney *U* test.

We employed a univariable logistic regression model analysis to assess the impact of the clinical parameters on the diagnosis of an LV thrombus upon CMRI. We then constructed a multivariable stepwise (forward elimination) model incorporating variables based on statistical significance in the univariate analysis (*p*-value < 0.05) and clinical relevance based on previous publications and clinical plausibility. Those variables that showed statistical significance (*p*-value < 0.05) in the multivariate analysis were retained in the final model and used to develop the ThrombScore. We applied a regression coefficient-based scoring method to construct a prediction rule for the development of LV thrombus (13, 14). The integer scores were assigned by dividing the risk-factor coefficients by the lowest coefficient and rounding up to the nearest unit to make a simple and user-friendly score. The discrimination ability of the model was evaluated using Harrel C-statistics and graphically displayed using ROC curves. The calibration of the model was examined using the Hosmer–Lemeshow Chi-squared statistic (15).

The analyzed variables had a missing data rate of <1% except for C-reactive protein (CRP) levels, which were available for 83% of the study cohort. To account for missing data and incorporate CRP in the multivariable analysis, our primary analyses were conducted on an imputed dataset where missing values were generated using multiple imputations by chained equations (MICE) (16). The imputed datasets were generated using the MICE package under the missing at random assumption. We applied the multiple imputation approach to create and analyze 100 multiply imputed datasets. A sensitivity analysis was also performed without the imputed data.

All statistical tests were two-sided, and a *p*-value of less than 0.05 was considered significant. Statistical analysis was performed using the SPSS statistical software 25.0.0 (IBM, Armonk, NY, USA) and R version 4.0.0 software (The R Foundation).

## Results

### Baseline characteristics

The study cohort comprised 337 consecutive STEMI patients without a prior history of coronary artery disease who further underwent CMRI during their index hospitalization. The median age was 59 years (IQR 51–66), and 302 (90%) of the participants were men. Overall, 34 (10%) patients were diagnosed with an LV thrombus upon CMRI. The baseline characteristics of the study cohort according to LV thrombus status are presented in Table 1. Patients with an LV thrombus did not show any statistically significant difference in the prevalence of cardiovascular risk factors, similar lipid profile, and blood counts upon hospital arrival, as compared with patients without an LV thrombus. In contrast, ECG characteristics significantly differed: patients with an LV thrombus had a higher ΣSTe upon presentation (13.3 ± 7.5 mm vs. 8.4 ± 6.2 mm, *p* = 0.001) and a higher prevalence of STe in the anterior leads (94% vs. 58%, *p* < 0.001), as compared with patients without an LV thrombus.

**Table 1:** Baseline characteristics of the study cohort upon hospital admission according to the left ventricular thrombus status.

	Total (*n* = 337)	No LV thrombus (*n* = 303)	LV thrombus (*n* = 34)	*p*-value
Age, mean ± SD, years	58.8 ± 11.1	58.6 ± 11	60.5 ± 12.4	0.389
Female, *N* (%)	35 (10.4)	31 (10.2)	4 (11.8)	0.767
Active smokers, *N* (%)	133 (39.5)	124 (41)	9 (26.5)	0.147
Hypertension, *N* (%)	119 (39.3)	110 (36.3)	9 (26.5)	0.243
Diabetes mellitus, *N* (%)	59 (17.5)	56 (18.5)	3 (8.8)	0.243
Dyslipidemia, *N* (%)	145 (43)	127 (42)	18 (53)	0.294
PVD, *N* (%)	5 (1.5)	5 (1.7)	0 (0)	1
Family history of CAD, *N* (%)	104 (31)	93 (30.7)	11 (32.4)	0.998
Prior ASA use, *N* (%)	40 (11.9)	37 (12.2)	3 (8.8)	0.781
Prior anti-coagulation use, *N* (%)	1 (0.3)	1 (0.3)	0 (0)	>0.9
Total cholesterol, mean ± SD, mg/dl	180 ± 39	180 ± 39	179 ± 37	0.9
HDL, mean ± SD mg/dl	42 ± 12	42 ± 12	43.6 ± 11.5	0.518
LDL, mean ± SD, mg/dl	120 ± 34	120 ± 39	121 ± 33	0.927
Triglycerides, mean ± SD, mg/dl	143 ± 93	1,145 ± 94	132 ± 77	0.389
WBC on admission, mean ± SD, K/µl	12 ± 78	14.6 ± 1.3	12.1 ± 3	0.899
Platelet count on admission, mean ± SD, K/µl	243 ± 66	243 ± 61	247 ± 99	0.809
MPV on admission, mean ± SD, fL	8.9 ± 1.2	8.9 ± 1.2	8.96 ± 1.1	0.899
Hemoglobin on admission, mean ± SD, g/L	14.6 ± 1.4	14.6 ± 1.4	14.9 ± 1.6	0.345
MCV on admission, mean ± SD, fL	87.9 ± 7.1	87.8 ± 7.4	88.9 ± 3.9	0.173
Sum of ST-segment elevation, mean ± SD, mm	8.9 ± 6.5	8.4 ± 6.2	13.3 ± 7.5	0.001
ST-segment elevation in anterior leads, *N* (%)	207 (61)	175 (58)	32 (94)	<0.001

ASA, acetyl salicylic acid; CAD, coronary artery disease; HDL, high-density lipoprotein; LDL, low-density lipoprotein; LV, left ventricular; MCV, mean corpuscular volume; MPV, mean platelet volume; PVD, peripheral vascular disease; SD, standard deviation; WBC, white blood cells.

### In-hospital characteristics

All patients underwent PPCI during their hospitalization. As shown in Table 2, patients with an LV thrombus were more frequently diagnosed with a low thrombolysis in myocardial infarction (TIMI) flow (0 or 1) upon coronary angiography (82% vs. 60%, *p* = 0.009). However, no difference was detected in the TIMI flow following PCI between the two study groups (*p* = 0.274). Notably, 27 (79%) patients with an LV thrombus had no resolution of STe on ECG post-revascularization, as compared with 85 (28%) patients without an LV thrombus (*p* < 0.001).

**Table 2:** Laboratory, echocardiographic, and angiographic characteristics of patients according to the left ventricular thrombus status.

	Total (*n* = 337)	No LV thrombus (*n* = 303)	LV thrombus (*n* = 34)	*p*-value
ST-segment elevation resolution, *N* (%)	225 (67)	218 (72)	7 (21)	<0.001
Highest tertile of Troponin-I, *N* (%)	115 (34)	93 (31)	22 (65)	<0.001
Peak CK, mean ± SD, IU/L	2,439 ± 2,408	2,178 ± 2,219	4,754 ± 2,796	<0.001
Peak CRP, mean ± SD, mg/L	47.2 ± 56.5	42.8 ± 54	86.6 ± 64.5	<0.001
TIMI flow at the start of the PCI, *N* (%):				0.009
0–1	209 (62)	181 (60)	28 (82)	
2–3	128 (38)	122 (40)	6 (18)	
TIMI flow at the end of the PCI, *N* (%):				0.274
0–1	3 (0.9)	2 (0.7)	1 (2.9)	
2–3	334 (99.1)	301 (99.3)	33 (97.1)	
The number of diseased coronary arteries, *N* (%):				0.170
0	4 (1.20	4 (1.3)	0 (0)	
1	179 (53)	155 (51)	24 (71)	
2	97 (29)	92 (30)	5 (15)	
3	57 (17)	52 (17)	5 (15)	
Left anterior descending artery stenting position, *N* (%):				<0.001
None	119 (35)	118 (39)	1 (3)	
Proximal	128 (38)	104 (34)	24 (71)	
Middle	86 (26)	78 (26)	8 (24)	
Distal	4 (1.2)	3 (1)	1 (3)	
GP IIb/IIIa inhibitors administration, *N* (%)	89 (26)	77 (25)	12 (35)	0.222
LVEF upon first echo, mean ± SD, %	43 ± 10	44 ± 10	35 ± 7	<0.001
LVEDD upon first echo, mean ± SD, cm	4.75 ± 0.48	4.76 ± 0.48	4.74 ± 0.49	0.827
LVESD upon first echo, mean ± SD, cm	3.2 ± 0.58	3.2 ± 0.6	3.07 ± 0.4	0.087
RWMA upon first echo, mean ± SD	1.71 ± 0.35	1.69 ± 0.36	1.89 ± 025	<0.001
LVEF upon second echo, mean ± SD, %	40 ± 9	41 ± 9	35 ± 7.3	<0.001
LVEDD upon second echo, mean ± SD, cm	4.82 ± 0.6	4.8 ± 0.6	4.9 ± 0.5	0.262
LVESD upon second echo, mean ± SD, cm	3.25 ± 0.72	3.22 ± 0.74	3.37 ± 0.6	0.291
RWMA index upon second echo, mean ± SD	1.74 ± 0.33	1.72 ± 0.33	1.83 ± 0.29	0.086

CK, creatine phosphokinase; CRP, C-reactive protein; LVEDD, left ventricular end-diastolic diameter; LVEF, left ventricular ejection fraction; LVESD, left ventricular end-systolic diameter; PCI, percutaneous coronary intervention; RWMA, regional wall motion abnormality; TIMI, thrombolysis in myocardial infarction score.

We detected a similar extent of CAD between the two study groups, as reflected by the number of diseased coronary vessels (*p* = 0.170). However, the proximal left anterior descending (LAD) artery was involved more frequently in patients with an LV thrombus (71% vs. 34%, *p* < 0.001).

Patients with an LV thrombus had significantly higher laboratory indices of myocardial infarction extent, including CK and TnI levels (*p* < 0.001 for both). In addition, patients with an LV thrombus had higher CRP levels (86.6 ± 64.5 vs. 42.8 ± 54, *p* < 0.001).

### Echocardiographic evaluation

All patients underwent at least one TTE assessment during hospitalization. Patients with an LV thrombus had a lower LVEF and a higher regional wall motion abnormality (RWMA) index. LV dimensions, as well as visually estimated right ventricular function, were similar between the two study groups (Table 2).

LV thrombus was identified in four (1.2%) patients upon the initial echocardiographic exam that was performed at a median period of 1 day (IQR 0–2). Of these, only two patients subsequently had a confirmed LV thrombus upon CMRI. Accordingly, the sensitivity of the first echocardiographic study in detecting LV thrombus was 6.25%, with a specificity of 99.3%, positive predictive value (PPV) of 50%, and a negative predictive value of 90%. Out of the 34 patients diagnosed with an LV thrombus upon CMRI, 30 (88%) patients underwent a second echocardiographic evaluation on day 4 ± 1 of the index event. The sensitivity of the second echocardiographic study to detect an LV thrombus was 59%.

### In-hospital complications

The rate of adverse events during hospitalization, including the insertion of an intra-aortic balloon pump (2.3% vs. 3%, *p*-value = 0.577), sustained ventricular arrhythmia (8.2% vs. 5.9%, *p*-value >0.9), or the development of high degree atrioventricular block (1% vs. 0%, *p*-value >0.9), was similar between the two study groups. None of the patients diagnosed with an LV thrombus developed stroke during the index hospitalization. Among patients being treated with non-vitamin K oral anticoagulant due to a concomitant diagnosis of either atrial fibrillation or venous thromboembolism (*N* = 15, 5%), LV thrombus was diagnosed in only one patient (6.7%). The length of hospitalization was significantly longer in patients with LV thrombus (5.2 ± 1.8 days vs. 7.8 ± 2.1, *p*-value < 0.001 for those without vs. with LV thrombus, respectively).

## Clinical predictors for LV thrombus development

Overall, 207 (61%) patients presented with anterior lead STe. Of these, 33 (15.9%) patients developed an LV thrombus. The vast majority of patients (*N* = 160, 77%) with STe in the anterior leads had at least a mildly reduced LVEF upon their initial TTE (EF < 45%). LV thrombus was detected in 29 (18%) patients with anterior STe and a mildly reduced LVEF.

The results of the univariate regression analysis are presented in Supplementary Table S1. The multivariate logistic regression model revealed that the lack of STe resolution (OR 4.4, 95% CI 1.78–11.2), maximal CK levels above the median (>1,704 IU/L) (OR 3.7, 95%, CI 1.2–11.4), STe in the anterior leads (OR 9.9, 95 CI 1.3–76.9), and maximal CRP levels above the median (≥24 mg/dl) (OR 3.09, 95% CI 1.2–8.2) were robust independent predictors for the presence of an LV thrombus (Table 3). Interestingly, after multivariable adjustment, both an LVEF < 45% and Troponin-I levels were no longer statistically significant (*p* = 0.66 and *p* = 0.7, respectively). The sensitivity analysis in patients with available CRP data (*N* = 280, 83%) revealed consistent results (Supplementary Table S2).

**Table 3:** Multivariable logistic regression analysis to predict the development of left ventricular thrombus.

	Odds ratio	95% Confidence interval	*p*-value
ST- segment elevation in anterior leads	9.9	1.3–76.9	0.028
Absence of ST-segment resolution	4.4	1.78–11.2	0.001
CRP above median (≥24 mg/L)	3.09	1.2–8.2	0.023
CK levels above median (>1,704 IU/L)	3.7	1.2–11.4	0.024

The model was constructed using forward elimination technique. Additional values that were included in the model and found to be not statistically significant: reduced left ventricular ejection fraction upon the first echo, highest tertile of Troponin-I, TIMI flow risk score on the beginning of primary percutaneous coronary intervention, and left anterior descending artery stenting. CK, creatinine phosphokinase; CRP, C-reactive protein; TIMI, thrombolysis in myocardial infarction score.

### ThrombScore development

**Figure 1:**
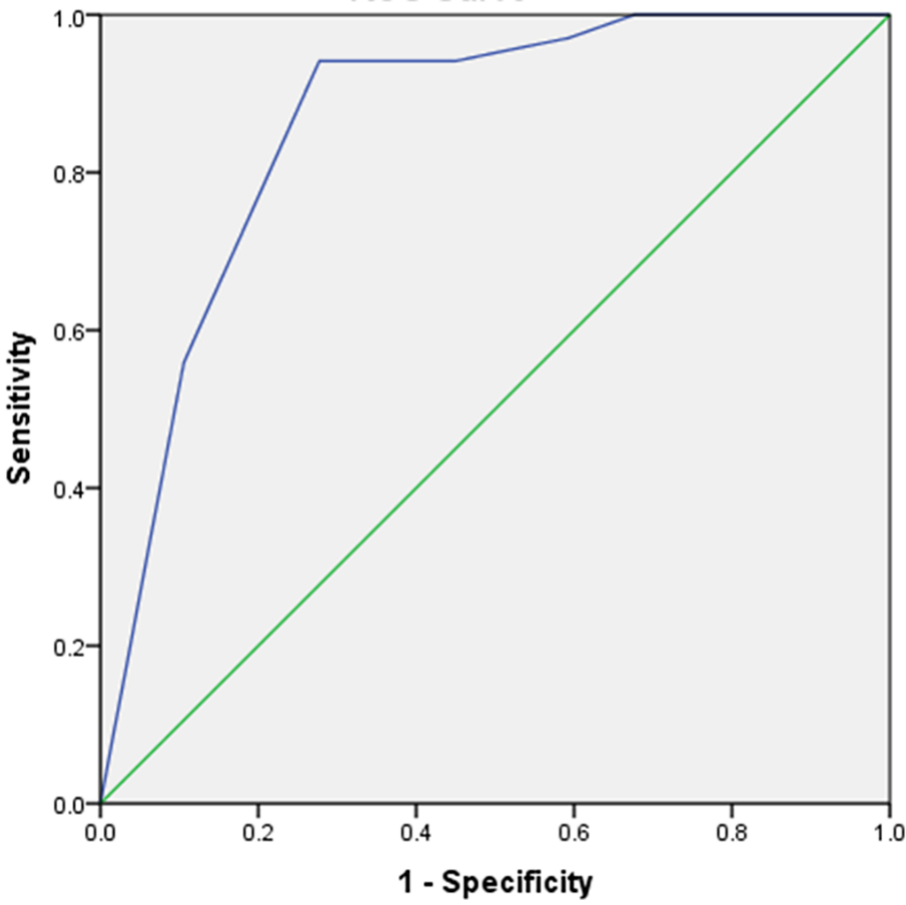
Receiver operator curve (ROC) of the ThrombScore model to predict left ventricular thrombus formation. The C-statistic of the model for the prediction of left ventricular thrombus was 0.86 (0.81–0.92; *p* < 0.001).

**Figure 2:**
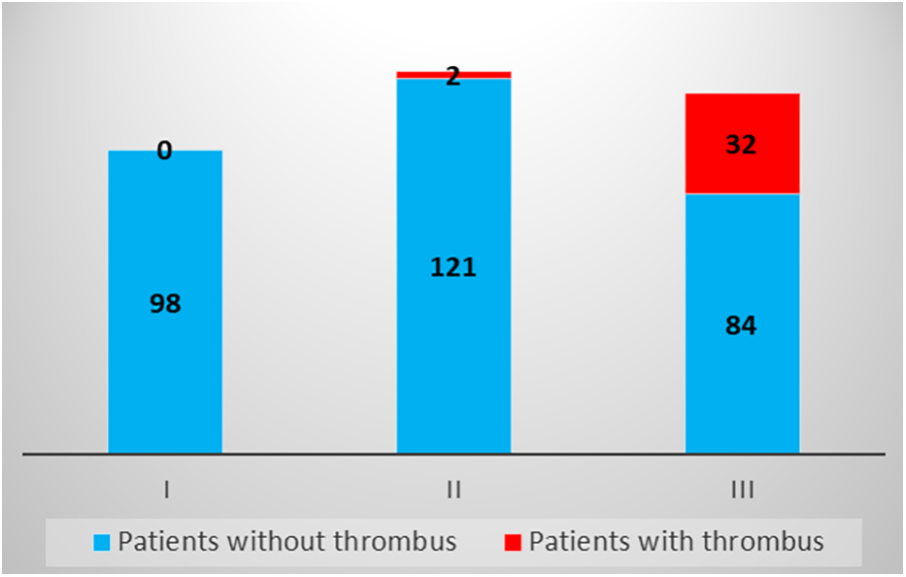
Risk of thrombus development according to the three-group ThrombScore. The proportion of patients diagnosed with a left ventricular thrombus gradually increased according to each score group: 0 (0%) for group I, 2 (1.6%) for group II, and 32 (27.6%) for group III (*p* for trend <0.001).

Based on the results of the multivariable logistic regression model, we constructed a simple six-point risk score to predict the development of an LV thrombus (Table 4). The odds ratio for thrombus development per one-point increase in the score was 3.2 (95% CI 2.1–5.01; *p* < 0.001). The model had good discriminative performance with c-statistics of 0.86 (0.8–0.92; *p* < 0.001) (Figure 1). Calibration was also good (Hosmer–Lemeshow goodness-of-fit chi-square 8.8; *p* = 0.12 for lack of fit). In order to simplify clinical utilization, we then transformed the score into three mutually exclusive risk categories: group I (0–1 points), group II (2–4 points), and group III (5–6 points). The prevalence of an LV thrombus gradually increased with each score group: 0% for group I, 1.6% for group II, and 27.6% for group III (*p* for trend <0.001) (Figure 2). The discriminative performance of the three-category model was comparable with the original six-point risk score with a c-statistic of 0.84 (0.79–0.89, *p*-value < 0.001) (Supplementary Figure S1). Similarly, the calibration of a three-category model was also good (Hosmer–Lemeshow goodness-of-fit chi-square 0.7; *p*-value = 0.79 for lack of fit).

**Table 4:** The ThrombScore components.

	Points
ST-segment elevation in the anterior leads 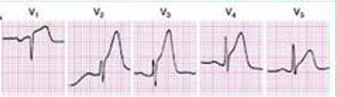	3
Lack of ST-segment resolution 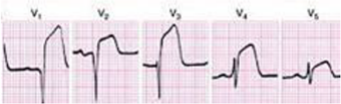	1
CRP above 24 mg/L 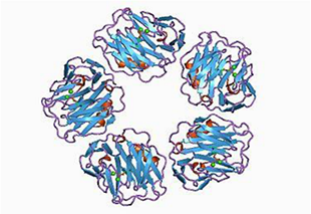	1
CK levels above 1,704 IU/L 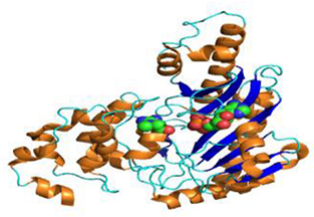	1

CK, creatinine phosphokinase; CRP, C-reactive protein.

## Discussion

The present study demonstrates that in a contemporary cohort of patients without a prior history of coronary artery disease, who present with a STEMI and were treated with PPCI, and subsequently underwent CMRI, approximately 10% developed an LV thrombus. Our findings suggest that the sole utilization of TTE is insufficient to rule out an LV thrombus in STEMI patients and that in some circumstances, CMRI is crucial in order to avoid a misdiagnosis of an LV thrombus. We developed a novel clinical score to predict the likelihood of developing LV thrombus in patients with STEMI in order to define those subsets of patients which will benefit most from further thorough evaluation using enhanced imaging modalities such as CMRI.

### Predictors for developing an LV thrombus

The pathophysiology of thrombus formation in post-infarction patients closely follows Virchow's triad: abnormal blood flow/stasis, hypercoagulability, and wall tissue injury (16–18). Consequently, clinical predictors could be classified according to Virchow's triad components. The most commonly described clinical risk factors for thrombus development are anterior wall MI and a reduced LVEF, emphasizing the impact of abnormal blood flow in Virchow's triad (3, 4, 19–21). Similarly, in a univariate model, we found that reduced LVEF, higher RWMA index, and LAD stenting were associated with LV thrombus formation. However, after multivariable adjustment, all these variables were no longer statistically significant. These results are not surprising as several studies pointed out that up to 20% of LV thrombi are not localized to the LV apex (22). With respect to LV function, Weinsaft et al. (23) described that only 12% of patients with thrombus had advanced systolic dysfunction (EF ≤ 30%), and only 18% had an LV aneurysm 

Wall tissue injury and hypercoagulability are interconnected in the context of acute MI: endothelial injury leads to the exposure of subendothelial tissue and collagen, which in turn results in a higher proinflammatory and prothrombotic state (18). Consequently, higher levels of cardiac biomarkers were found to be associated with an increasing rate of LV thrombus formation (24). Furthermore, the lack of ST resolution despite prompt reperfusion seems to be a powerful predictor of LV thrombus development, probably reflecting more intensive subendocardial damage and microvascular obstruction (25). Lastly, inflammatory markers such as CRP, neutrophil to leucocyte ratio, and interleukin-6 levels were previously found to be related to LV thrombus development (26). In the current study, elevated CRP levels were indeed independently associated with LV thrombus formation.

### Timing of echocardiographic evaluation

Despite its widespread availability, TTE has a rather low sensitivity of approximately 21%–35% for LV thrombus detection (5–7, 23). In the current study, the vast majority of patients (85%) diagnosed with an LV thrombus underwent two subsequent TTE exams during hospitalization. We found that the sensitivity of the second TTE was significantly higher, as compared with that of the first exam (59% vs. 6.25%), approaching the reported sensitivity of contrast TTE according to one study (23). This is due to several issues: first, the timing of imaging has diagnostic relevance. LV thrombi were identified within the first 24 h in a minority of patients (27). Moreover, Meurin et al. (28) found that 25% of LV thrombi were identified when TTE was performed during the first week while an additional 38% were identified when TTE was repeated during the second week post-MI. Second, as the second TTE was done at the discretion of the treating physician, we cannot exclude the possibility that this was performed in patients with a higher clinical suspicion for developing an LV thrombus and, therefore, resulted in a higher detection rate. Nevertheless, a considerable proportion of LV thrombi have been missed, even upon a repeated dedicated TTE performed during hospitalization.

### ThrombScore

The recently published scientific statement of the American Heart Association (AHA) recommends to perform CMRI in cases where TTE is suggestive but non-diagnostic, or when a clinical concern remains despite a negative TTE. However, no definition was provided for high-risk patients for thrombus development (29). Given the limited sensitivity of TTE to detect an LV thrombus and the high cost and limited availability of CMRI, clinical risk scores are warranted to accurately identify those patients with a high pre-test probability for the detection of an LV thrombus. Equally significant is the need to identify patients in whom an LV thrombus could be safely ruled out using TTE. Several diagnostic strategies were proposed to identify patients who are at the highest risk of developing an LV thrombus. Weinsaft et al. (23) showed that an apical wall motion score has a sensitivity of approximately 100% for detecting an LV thrombus. Notably, all thrombi in this cohort were apical in location, and the performance of this score might be less accurate in cases of thrombi located in other areas. Similarly, a recently proposed algorithm suggests to utilize TTE with contrast in all STEMI patients within the first 24 h and to perform a repeat exam within 72 h in those with high-risk features, such as apical akinesis, a high apical wall motion score, or the inability to visualize the apex. In patients with unequivocal finding on the second TTE, additional imaging with CMRI or computed tomography is recommended (18). Nevertheless, all these strategies focus on the detection of LV thrombi in the apical location, which could result in underdiagnoses of non-apically located thrombi.

**Figure 3:**
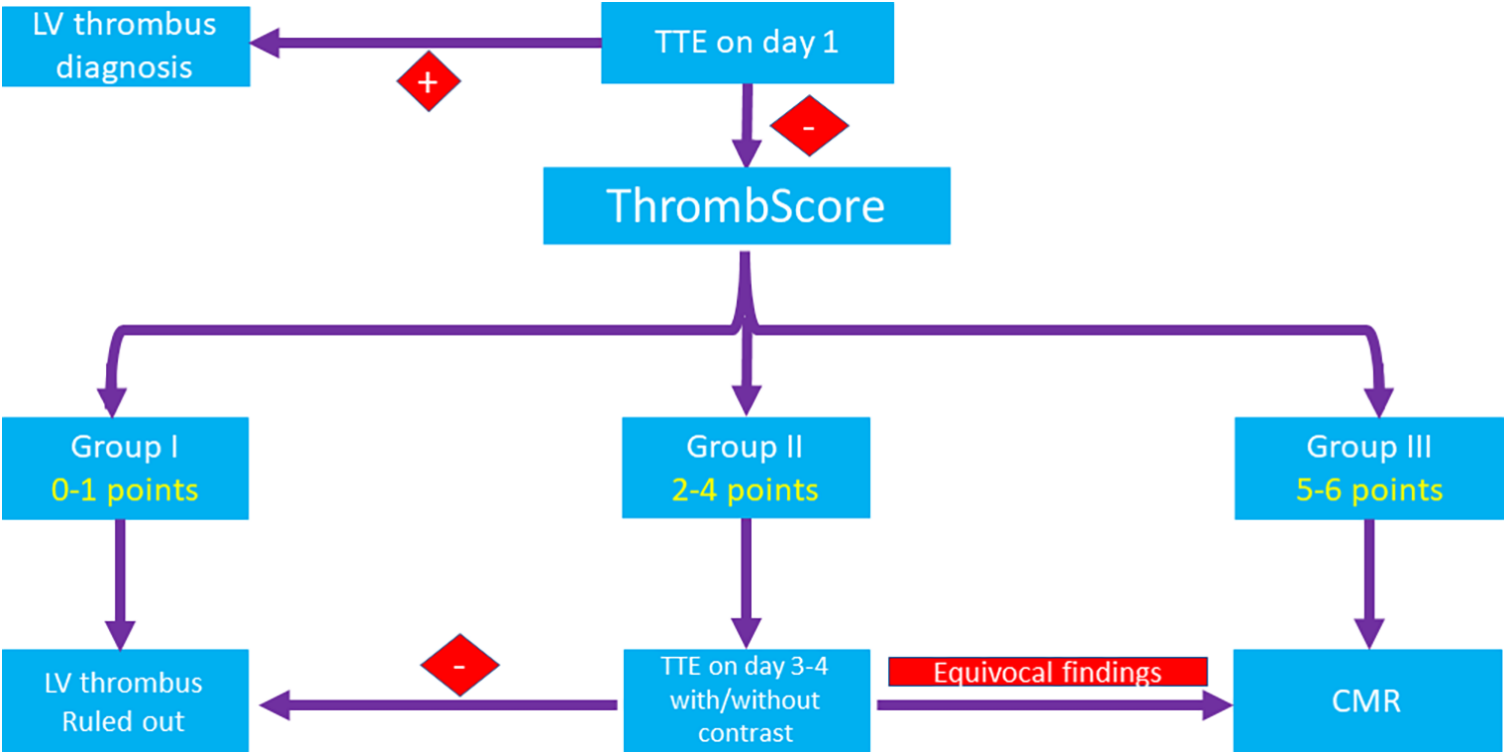
A proposed algorithm for the diagnosis of an LV thrombus. In patients within the lowest (I) category (0–1 points), an LV thrombus can be safely ruled out by TTE only. In patients within the highest (III) category (5–6 points), CMRI is recommended to rule out an LV thrombus when it is not detected by TTE. Patients within the intermediate (II) category (2–4 points) should probably undergo re-evaluation with TTE with or without contrast and be considered for imaging by CMRI on an individual basis. CMRI, cardiac magnetic resonance imaging; CK, creatinine phosphokinase; CRP, C-reactive protein; LV, left ventricular; STe, ST-segment elevation; STEMI, ST,-segment elevation myocardial infarction; TTE, transthoracic echocardiography.

Thus, we constructed a novel score that addresses the different components of Virchow's triad. This score incorporates simple and available parameters such as baseline ECG and baseline blood tests and allows to classify the patients at the very early stage of their hospitalization. Notably, although anterior STe on ECG has the highest impact on patients' risk according to our findings, it is insufficient to classify a patient to the highest-risk category based solely on this finding. According to our findings, for patients within the lowest I category (0–1 points), an LV thrombus can be safely ruled out by relying solely on the basis of TTE (Figure 3). On the other hand, in patients within the highest III category (5–6 points), enhanced imaging techniques such as CMRI is needed to rule out an LV thrombus when it is not detected by TTE, as it may not be sensitive enough for these patients, even if it is repeated later during hospitalization. Based on this strategy, only 34% of patients in our study were classified at the highest-risk group and hence should be referred for CMRI, compared with 48% of patients who should be referred for CMRI based only on the presence of an anterior wall MI and a reduced LVEF (<45%). Patients within the intermediate II category (2–4 points) should probably undergo re-evaluation with a repeat TTE/contrast TTE and be considered for imaging by CMRI on an individual basis.

## Limitations

Our study has several limitations. First, because this is a single-center, retrospective analysis, the generalizability of our findings may be limited. Second, our cohort is modest in size, and the overall number of thrombi limits the power of the multivariable analysis. We cannot compare our findings with contrast echocardiography because we do not use it on a regular basis. Third, left ventricular diameters rather than volumes were calculated and incorporated into the multivariable regression model. Finally, the model we developed was not validated using an external cohort and thus serves only to generate hypotheses.

## Conclusion and clinical implication

The presence of an LV thrombus is not negligible in STEMI patients. Relying solely on TTE is insufficient to exclude an LV thrombus in high-risk patients who require advanced imaging. Using a rather simple score such as the ThrombScore during the early stage of hospitalization may aid in identifying the patients who are at a higher risk of developing an LV thrombus and require more advanced imaging in order to better stratify them. Further prospective studies are needed to validate and generalize our findings.

## Data Availability

The data underlying this article will be shared on reasonable request to the corresponding author.
