# Supplementary material for: Clinical predictors of left ventricular thrombus after myocardial infarction as detected by magnetic resonance imaging
Source: Front Cardiovasc Med. 2024 Jan 16;10:1275390. doi: 10.3389/fcvm.2023.1275390 (PMC10824980; doi:10.3389/fcvm.2023.1275390)
Supplement: Supplementary file 1 [file Table1.docx]

**Supplementary Tables**

**Supplementary Table S1. Univariable logistic regression analysis for thrombus formation.**

|  | Odds Ratio | 95% Confidence Interval | P value |
| --- | --- | --- | --- |
| Age (per 1-year increment) | 1.02 | 0.98-1.05 | 0.337 |
| Female gender | 1.17 | 0.38-3.54 | 0.781 |
| Active smoker | 1.92 | 0.87-4.26 | 0.107 |
| Diabetes Mellitus | 2.34 | 0.69-7.9 | 0.171 |
| Prior ASA use | 0.69 | 0.20-2.39 | 0.564 |
| Pain onset to wire crossing, per 1 hour increment | 1.005 | 0.98-1.03 | 0.62 |
| Sum of ST segment elevation>7 mm | 5 | 2.1-11.9 | <0.001 |
| ST-segment resolution | 9.9 | 4.1-23.6 | <0.001 |
| ST-segment elevation in anterior leads | 24.1 | 3.2-178.8 | 0.002 |
| LVEF on 1^st^ echo^a^ | 0.9 | 0.86-0.94 | <0.001 |
| LVEF below 45% on 1^st^ echo | 7.1 | 2.4-2.5 | <0.001 |
| RWMA on 1^st^ echo | 5.6 | 1.85-17.1 | 0.002 |
| LVEF on 2^nd^ echo | 0.92 | 0.88-0.97 | 0.003 |
| RWMA on 2^nd^ echo | 2.99 | 0.79-11.3 | 0.106 |
| Maximal CRP level, per 0.1 mg/L increment | 1.01 | 1.005-1.01 | <0.001 |
| CRP above median (24 mg/L) | 5.6 | 2.25-13.9 | <0.001 |
| Highest TnI tertile | 4.14 | 1.97-8.7 | <0.001 |
| CK levels | 1.0003 | 1.0002-1.0005 | <0.001 |
| CK above median | 8.9 | 3.1-26.1 | <0.001 |
| TIMI flow 0-1 pre intervention, | 3.15 | 1.27-7.82 | 0.014 |
| TIMI flow 0-1 post intervention | 4.56 | 0.4-51.7 | 0.220 |
| GP IIb/IIIa inhibitors administration | 1.6 | 0.76-3.34 | 0.218 |

^a^LVEF as continuous variable

ASA = acetyl salicylic acid; CK = creatine phosphokinase; CRP = C reactive protein; IABP = intra-aortic balloon pump, LVEDD = left ventricular end diastolic diameter; LVEF = left ventricular ejection fraction; LVESD = left ventricular end systolic diameter; ; RWMA = Regional wall motion abnormality.

**Supplementary Table S2. Multivariable logistic regression analysis to predict Left Ventricular thrombus development in patients without imputed CRP data (N=280).**

|  | Odds Ratio | 95% Confidence Interval | P value |
| --- | --- | --- | --- |
| ST- segment elevation in anterior leads | 7.4 | 0.93-58.4 | 0.058 |
| Absence of ST-segment resolution | 5.3 | 1.9-15.3 | 0.002 |
| CRP above median (>43 mg/dL) | 2.5 | 1.02-6.2 | 0.045 |
| CK levels above median (>1704 IU/L) | 3.2 | 1.01-10.2 | 0.048 |

The model was constructed using forward elimination technique. Additional values that were included in the model and found to be not statistically significant: left ventricular ejection fraction upon the first echo, highest tertile of Tropinin I, TIMI flow risk score on the start of primary PCI, left anterior descending artery stenting.

CK - creatinine phosphokinase, CRP- C-reactive protein.

**Supplementary figures**

**SF1. Receiver Operator Curve of the 3-group ThrombScore model to predict Left ventricular thrombus formation**


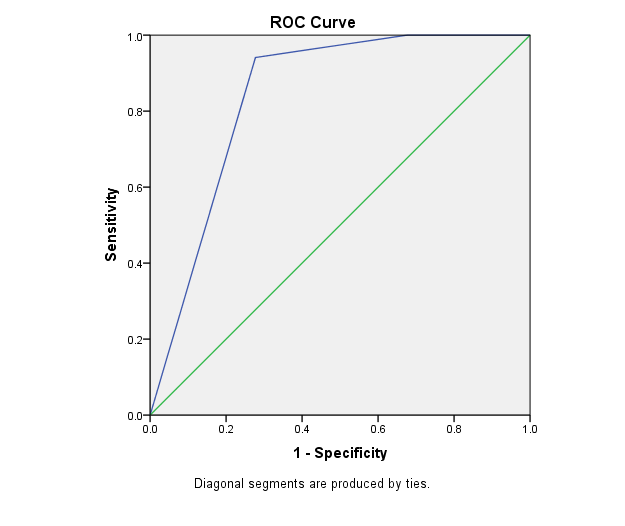


C-statistics of the model for prediction of left ventricular thrombus was 0.84 (0.79-0.89, p value < 0.001).
